# Systemic doxycycline as an adjunct to scaling and root planing in diabetic patients with periodontitis: a systematic review and meta-analysis

**DOI:** 10.1186/s12903-019-0873-7

**Published:** 2019-09-05

**Authors:** Kenneth Chou Hung Yap, Shaju Jacob Pulikkotil

**Affiliations:** School of Dentistry, International Medical University, 126, Jalan Jalil Perkasa 19, Bukit Jalil, Kuala Lumpur, Malaysia

**Keywords:** Dental scaling, Diabetes, Doxycycline, Periodontitis, Root planing, Systematic review

## Abstract

**Background:**

To compare the effectiveness of systemic doxycycline as an adjunct to scaling and root planing (SRP) with SRP alone in improving periodontal clinical attachment level and glycemic control in diabetic patients with periodontitis.

**Methods:**

Two independent reviewers (KY and SJ) screened two electronic databases, PubMed and Scopus, for randomized clinical trials on the use of systemic doxycycline as an adjunct to scaling and root planing in improving periodontal status and glycemic control in diabetic patients with periodontitis using predetermined selection criteria within a 3-month period. The reviewers independently did data screening, data selection, data extraction and risk of bias. Quality of studies involved was analysed using the revised Cochrane Risk of Bias 2.0. Weighted standard mean differences (SMD) and 95% confidence intervals were calculated using a random effects meta-analysis model. Publication bias was evaluated using funnel plot. Quality of evidence was evaluated by Grading of Recommendations Assessment, Development and Evaluation (GRADE) approach.

**Results:**

Electronic searches provided 1358 records and six studies were selected. The meta-analyses indicated that there was no statistically significant difference in the improvement of periodontal status with the use of systemic doxycycline as an adjunct for scaling and root planing (SRP). SMD of clinical attachment levels (− 0.22 [− 0.52, 0.08]) and HbA1c levels (− 0.13 [− 0.41, 0.15]) were calculated. Overall risk of bias is high in 2 out of 6 studies involved.

**Conclusion:**

Systemic doxycycline when used in addition to scaling and root planing yields no significant improvement of clinical attachment levels for periodontal status and reduction of HbA1c levels in treatment of diabetic patients with periodontitis when comparing the test group to the control group.

## Background

Periodontitis is a chronic inflammatory disease which leads to progressive destruction of the periodontium and tooth loss [1]. Removal and control of the accumulation of the plaque biofilm is the mainstay of periodontal treatment [2]. Scaling and root planing (SRP) is the mechanical removal of plaque, calculus and diseased cementum. Some patients might still encounter constant loss of attachment because of the failure of scaling and root planing to reduce or eliminate periodontal microorganisms to achieve health even after thorough SRP [3]. Some bacteria are likely to get away from host defenses after non-surgical periodontal therapy due to restricted means of entry to the root surface and the tissue-invading abilities of the pathogens [4]. This raises the question of the effectiveness of SRP due to the bacteria residue in the tissues and also the inadequate plaque control of each patient [5]. To battle this phenomenon, a variety of antibiotics were used as adjunctive therapy to enhance the treatment outlook of SRP [6]. Systemic doxycycline have been long used as an adjunct to supplement the effect of scaling and root planing [7].

A substantially larger increase in CAL in diabetic patients with periodontitis who were given systemic doxycycline were seen as compared to without doxycycline after 3 months [8, 9]. These outcomes shows that there is a better resolution of periodontal lesions when patients are given systemic doxycycline as compared to just SRP. On the contrary, other studies showed results that are contradictory to the previous results stating that there is no significant increase in CAL in the group that has received SRP with systemic doxycycline as compared to the group of patients treated with SRP after 3 months [10]. In terms of HbA1c levels, some studies suggested that there is a marked reduction in HbA1c levels in group with SRP with systemic doxycycline as compared to the control group which only had SRP. On the other hand, Promsudthi et al. and O’Connell et al. was not able to show a marked reduction in HbA1c levels when test group (SRP with doxycycline) and control group (SRP only) were compared to each other after 3 months [9, 10]. There is a need to identify the efficacy of the systemic doxycycline in improving the periodontal health and glycemic control in diabetic patients with periodontitis given the lack of concrete evidence to show improved metabolic control and periodontal status when systemic doxycycline is used as an adjunct to scaling and root planing. This review based on a systematic search will identify the eligible studies and analyse data to determine the efficacy of systemic doxycycline as an adjunct to scaling and root planing in diabetic patients with periodontitis.

## Methods

This review reported based on PRISMA guidelines and was registered in PROSPERO database (CDR42018103828). This is a systematic review of randomized clinical trials that evaluate differences in clinical attachment levels (CAL), pocket depth (PD), bleeding on probing (BOP) and glycated hemoglobin (HbA1c) levels in diabetic patients with periodontitis after scaling and root planing (SRP) with systemic doxycycline in comparison to just SRP alone.

### Research question according to PICOS

What is the difference in the effect on the periodontal and glycemic status (O) with systemic doxycycline as an adjunct to scaling and root planing (SRP) (I) as compared to SRP alone (C) in diabetic patients with periodontitis (P) from randomized controlled trials (S)?

### Study selection

Two electronic databases, Pubmed and Scopus, were searched using suitable keywords in various combinations until April 2018 to identify randomized controlled trails (RCTs) that compared systemic doxycycline as an adjunct to SRP (test group) to SRP alone (control group) in diabetic (type 1 or 2) patients with periodontitis.

### Inclusion criteria

The subsequent inclusion criteria were used for the selection of studies:

- Randomized clinical trials;
- patients diagnosed with periodontitis and diabetes mellitus.
- interventions assessing the effectiveness of systemic doxycycline as an adjunct to SRP.
- studies reporting one or more clinical periodontal parameters as outcome including pocket depth (PD), or clinical attachment level (CAL)
- studies reporting metabolic parameter such as HbA1c before and after systemic doxycycline application and;
- studies published in English language only.

### Exclusion criteria

The exclusion criteria included.

- in vitro and experimental studies.
- ex vivo studies.
- case reports.
- animal studies.

### Data extraction

Titles and abstracts of articles that satisfy the selection protocol were screened independently by two reviewers (KY and SJ) with discord solved by discussion. Following selection of studies, data extraction was done on patient characteristics, disease characteristics, periodontal attachment outcome data in form of clinical attachment level (CAL), probing depth (PD), bleeding on probing (BOP) and glycemic status (HbA1c levels) with CAL and HbA1c levels being the primary outcome measurements for periodontal status and glycemic status respectively. Data extracted from the eligible studies were independently by two reviewers (KY and SJ) and disagreement resolved through discussion.

### Risk of bias assessment

Risk of Bias assessment was done using the revised Cochrane tool 2.0 for Risk Of Bias (ROB) was done for all studies independently by two reviewers (KY and SJ) and consensus reached on disagreement.

### Statistical analysis

Meta-analysis was done using Review Manager 5.3. Primary meta-analysis using random effects model was done for eligible RCTs on CAL, PD, BOP and HbA1c levels. Subgroup analysis was done based on the patient characteristics identified from the studies. Sensitivity analysis based on risk of bias on the included studies. Publication bias was assessed through visualization of the funnel plot was done. Quality of evidence and confidence in estimates was assessed using GRADE Working Group criteria which was done using GRADE development tool using the method of assessing the certainty in evidence (also known as quality of evidence or confidence in effect estimates) and the strength of recommendations in health care.

## Results

**Fig. 1:**
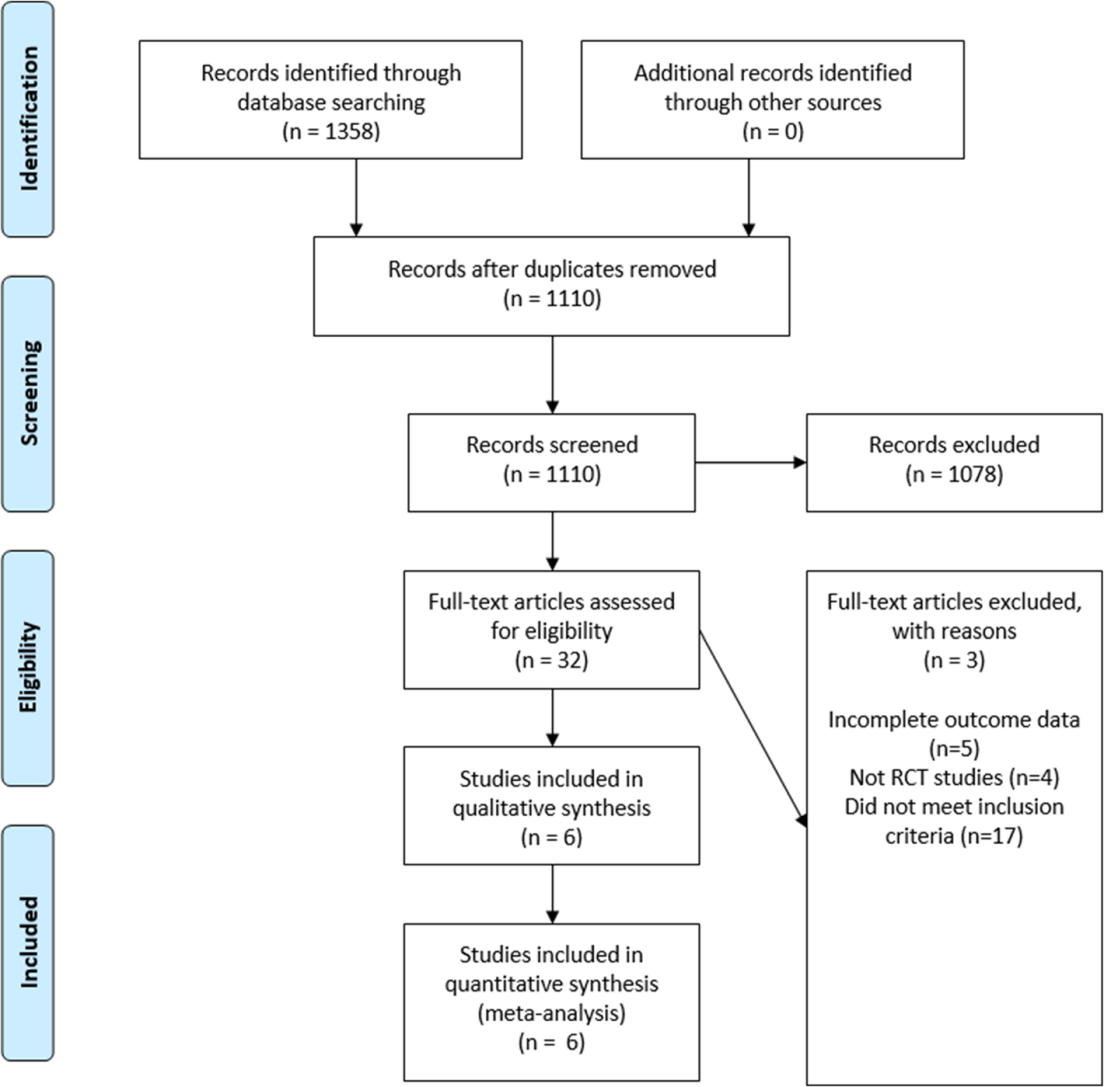
PRISMA flow chart

**Fig. 2:**
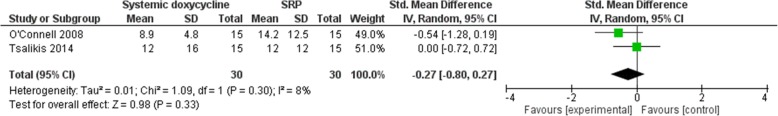
3 Month bleeding on probing (BOP)

**Fig. 3:**
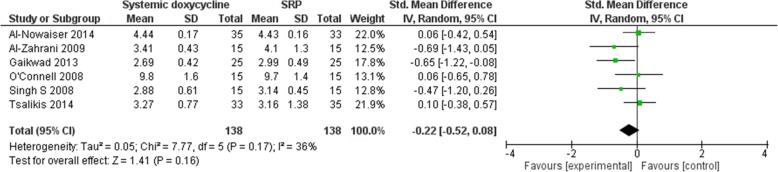
3 Month clinical attachment levels (CAL)

**Fig. 4:**
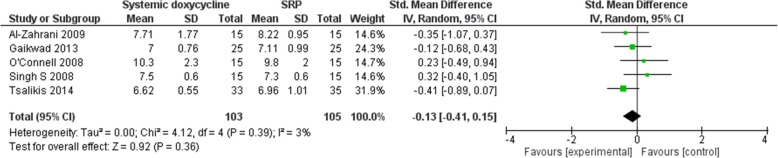
3 Month glycated hemoglobin (HbA1c)

**Fig. 5:**
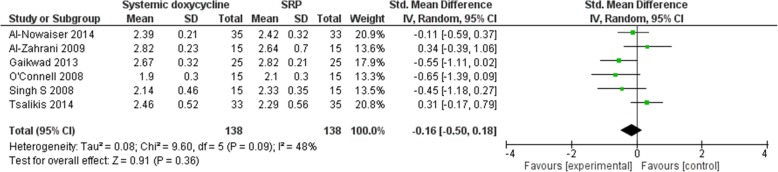
3 month probing depth (PD)

The electronic search retrieved 1358 records. No other additional records were identified through other sources. After removing the duplicates, there were 1110 records remaining, of which 1078 of them did not meet the inclusion criteria. After that, 32 of the remaining articles were assessed for eligibility. From these 32 studies, 26 of the studies did not meet the inclusion criteria and 6 of the studies were included in the meta-analyses. Figure 1 shows the studies that have been found with duplicates removed, screened and assessed for eligibility. An in-depth analyses of all studies showed low-to-moderate heterogeneity [(BOP: *P* = 0.30, I_2_ = 8% (Fig. 2); CAL: *P* = 0.17, I_2_ = 36% (Fig. 3); HbA1c: *P* = 0.39, I_2_ = 3% (Fig. 4); PD: *P* = 0.09, I_2_ = 48% (Fig. 5)]. All 6 studies had adequate data for the statistical analysis of CAL gain and HbA1c reduction at 3 months but insufficient data at 6 months and above. Total number of patients included are 276 at 3 months. Forest plots of primary and secondary data outcomes are given in Figs. 2, 3, 4 and 5. Meta-analysis showed no statistically significant improvement in CAL at 3 months (SMD -0.22; − 0.52, 0.08) and HbA1c levels at 3 months (SMD -0.13; − 0.41, 0.15) when systemic doxycycline is used as an adjunct to scaling and root planing compared to just SRP alone. It is the same case in PD (SMD -0.16;-0.50, 0.18) and BOP (SMD -0.27; − 0.80, 0.27) where there is no significant improvement when systemic doxycycline is used as an adjunct to scaling and root planing compared to just SRP alone after 3 months. This shows that there is no difference in effectiveness of systemic doxycycline as compared to control group.

**Fig. 6:**
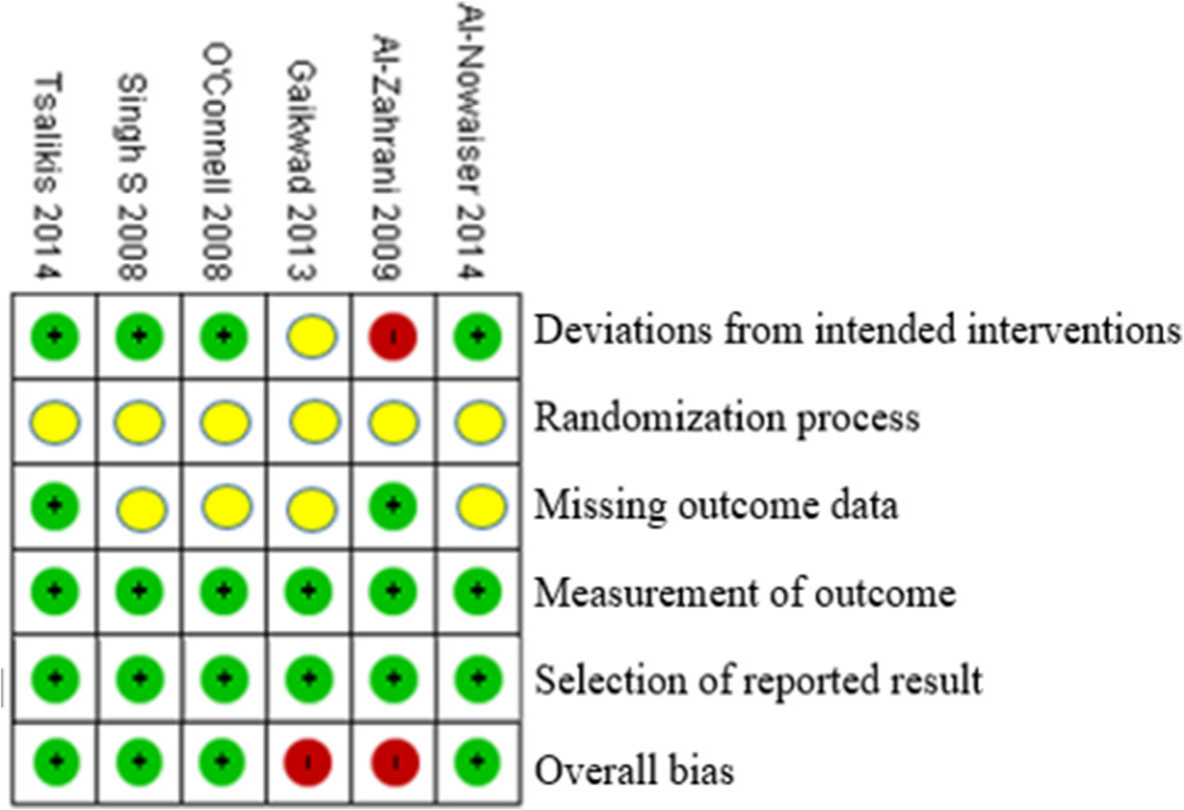
Assessment of risk of bias. Green: low risk of bias, Yellow: Moderate risk of bias, Red: High risk of bias

Figure 6 provides information about the risk of bias of the studies that are included. From the tabled data, none of the 6 studies taken had a low risk of bias in any of the domains that were assessed. All 6 studies were reviewed by each of the reviewers independently and came up with the conclusion that only 2 out of 6 of the studies taken are at a high risk of bias, mainly Gaikwad 2013 and Al-Zahrani 2009.

**Fig. 7:**
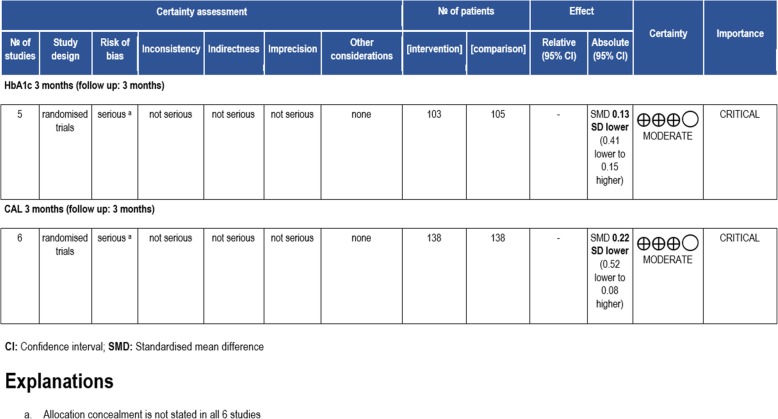
GRADE working criteria

Figure 7 shows GRADE Working Criteria which is used to assess the standard of the scientific research in systematic reviews [12]. Risk of bias of HbA1c levels and CAL data after 3 months was found to be serious due to the lack of information regarding allocation concealment in all 6 of the studies that were reviewed.

The general characteristics of all the 6 studies are shown in Table 1. Some of the studies used different dose and duration of dose given to the patient.

**Table 1:** Characteristics table

Study	Year	Gender & number (n)	Country	Number of included patients (n)	Age range/mean (years)	Periodontitis type	Probing Method	Intervention	Study duration (months)
Singh et al.	2008	Not indicated	India	Test (15) Control (15)	Not indicated	Moderate to severe	William’s periodontal probe, sites not stated	Test = Full mouth SRP + doxycycline 100mg, 2 tablets taken on 1^st^ day, followed by 1 tablet daily for 14 days Control = Full mouth SRP	3
O’Connell et al.	2008	Female – 16 Male – 14	Brazil	Test (15) Control (15)	52.9	Chronic	Computerized periodontal probe, 6 sites	Test = Full mouth SRP + doxycycline 100mg, 2 tablets taken on 1^st^ day, followed by 1 tablet daily for 14 days Control = Full mouth SRP	3
Al-Zahrani et al.	2009	Female – 18 Male – 11	Saudi Arabia	Test (15) Control (14)	52.28	Moderate to severe chronic	6 sites	Test = Full mouth SRP + doxycycline 100mg, 2 tablets taken on 1^st^ day, followed by 1 tablet daily for 14 days Control = Full mouth SRP	3
Tsalikis et al.	2014	Female – 28 Male – 38	Greece	Test (31) Control (35)	60.42	Chronic	Automated Florida probe, 6 sites	Test = Full mouth SRP + doxycycline 100mg, 2 tablets taken on 1^st^ day, followed by 1 tablet daily for 20 days Control = Full mouth SRP	3, 6
Gaikwad et al.	2013	Female – 16 Male – 34	India	Test (25) Control (25)	30–70	Chronic generalized	UNC 15 probe, 4 sites	Test = Full mouth SRP + doxycycline 100mg 1 tablet daily for 15 days Control = Full mouth SRP	1, 2, 3, 4
Al-Nowaiser et al.	2014	Female – 26 Male – 42	Saudi Arabia	Test (35) Control (33)	42	Chronic	manual Florida periodontal probes, 6 sites	Test = Full mouth SRP + doxycycline 100mg 1 tablet daily for 15 days Control = Full mouth SRP	3

## Discussion

Results of 3-month Bleeding On Probing (BOP), Clinical Attachment Levels (CAL), Pocket depth (PD) and HbA1c levels are taken due to the limited number of studies done for 6 months and above for each of these clinical parameters. Systematic reviews like Grellmann AP et al. showed that the use of doxycycline did not significantly improve probing depths whereas Ronaldo Lira Junior et al. and Wang-Tze Fang et al. showed that adding doxycycline to periodontal therapy with SRP does not significantly improve metabolic control in patients with type 2 diabetes mellitus and chronic periodontitis [13–15]. Although systemic doxycycline inhibits metalloproteinase activity and also has antimicrobial effects which helps in reducing inflammation of periodontal tissues [15], HbA1c levels did not statistically improve after 3 months after administration of systemic doxycycline as an adjunct to SRP in our study. In terms of periodontal status, CAL, PD and BOP generally showed no significant improvement when systemic doxycycline is used as an adjunct to SRP, although 1 study showed an a favorable increase in CAL when systemic doxycycline is used [16]. On the other hand, another study revealed a significant improvement in probing depths when systemic doxycycline is used as an adjunct to SRP [7]. This study that we have done also proves the current systematic reviews that systemic doxycycline as an adjunct to scaling and root planing (SRP) in diabetic patients with periodontitis does not show any benefit versus SRP alone in boosting metabolic control (HbA1c) as well as periodontal status in terms of clinical attachment levels (CAL) after 3 months of treatment.

The incorporation of doxycycline in SRP can have a higher chance to alter the pathogenic bacterial group, thus making it a more favorable environment for stable recolonization in the long run in gingival pockets which are recently scaled. This consequently creates a stable biofilm community which can also be found in individuals with no periodontal disease [17–19]. Nevertheless, bacterial resistance is an issue when there is broad usage of systemic antibiotics such as doxycycline used in periodontics [20]. Therefore, every clinician should weigh the pros and cons of prescribing these antibiotics to prevent bacterial resistance and other undesirable side effects of antibiotics. At this moment of time, about 75% of patients with chronic periodontitis showed that periodontal bacteria such as Aggregatibacter Actinomycetemcomitans are now resistant to at least one of the common antibiotics which includes doxycycline [21]. Thus, their prescription is only considered in clinical situations where the benefits of prescribing outweighs the undesired effects of antibiotics.

As shown in our study, there is no significant difference in CAL when systemic doxycycline is used as an adjunct to SRP and borderline statistical difference for PD. Although CAL is the gold standard for diagnosis of periodontitis, probing depths are also as important as CAL because the presence of deep pockets increases the risk of development of periodontal disease which will signals the need for more treatment [22, 23]. Studies from Sokransky and Haffajee et al. have demonstrated that more bacteria are found in deep pockets [17]. Increase in PD shows inflammatory changes, and clinical attachment levels will increase when inflammation of periodontium decreases and long junctional epithelium starts to develop [24].

Wang et al. have evaluated the efficacy of SRP with systemic doxycycline on reduction of HbA1c in diabetic patients in which 3 trials were included in the meta-analysis and there was no significant change in HbA1c levels by using systemic doxycycline as an adjunct to SRP [25]. As for Ronaldo Lira Junior et al’s study, they had 3 extra trials and found results complementing Wang et al’s study, proving that systemic doxycycline is not beneficial when used as an adjunct to SRP over SRP alone on in terms of reducing HbA1c levels [14]. These systematic reviews and meta-analyses are similar to our study which also shows that systemic doxycycline showed no significant benefits when used as an adjunct to SRP as compared to just SRP alone [− 0.13 (− 0.41, 0.15)].

In terms of heterogeneity of the studies involved, the hetereogeneity percentage for BOP, CAL, HbA1c and PD are 8, 36, 3 and 48% respectively. According to Cochrane, 0–40% indicates low heterogeneity and 40–60% indicates moderate heterogeneity [29]. Results BOP and HbA1c are categorized as low heterogeneity, whereas CAL has a low to moderate heterogeneity. On the other hand, PD is categorized as moderate heterogeneity. The moderate heterogeneity of PD might be due to difference in dose and duration of systemic doxycycline as stated in the characteristics table in Table 1. However, since the primary outcomes are CAL and HbA1c which are calculated as low heterogeneity, it would not affect the results of the meta-analysis significantly.

From the GRADE assessment, certainty of the results of this meta-analysis is moderate which indicates that further research is will most probably have a major effect on our confidence intervals in the estimate of effect and may change the estimate. Certainty was moderate due to the moderate overall risk of bias in the 6 studies involved. The importance of this meta-analysis results is stated as “critical” because this systematic review aims to avoid the excessive use of systemic doxycycline without any tangible benefit, reducing the risk of antibiotic resistance against systemic doxycycline in the long run.

**Fig. 8:**
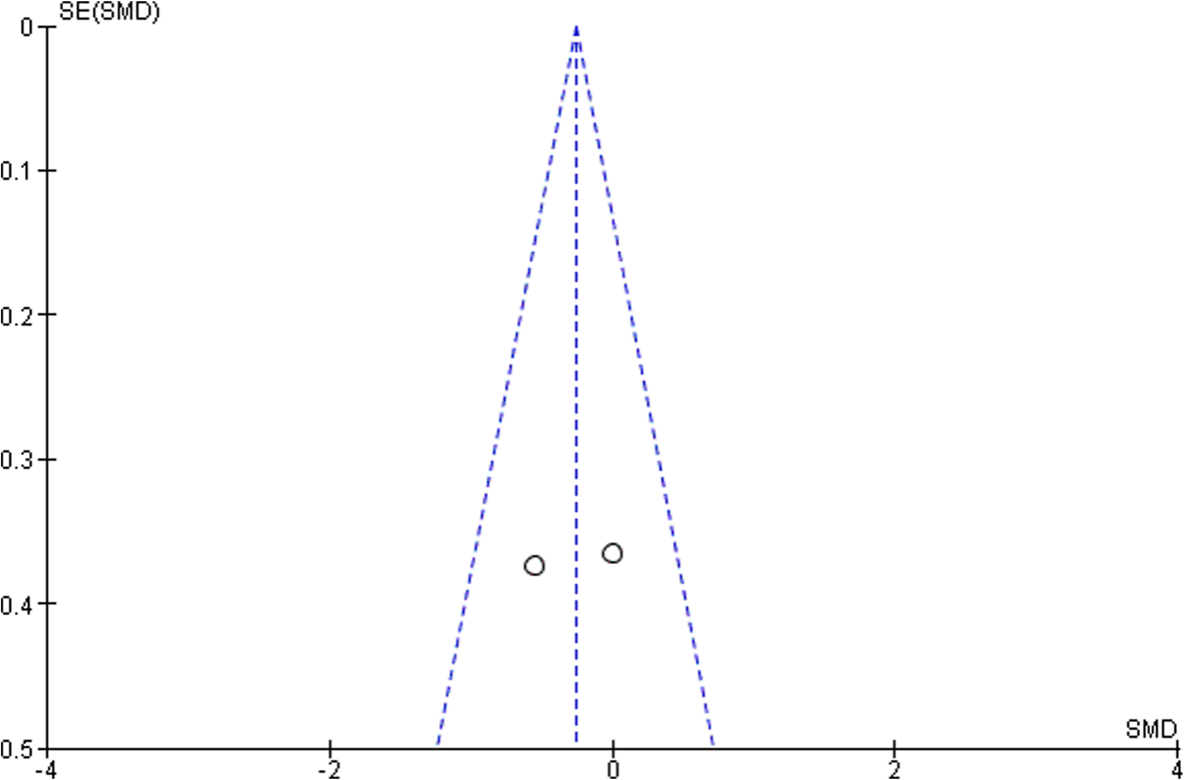
3 Month bleeding on probing (BOP)

**Fig. 9:**
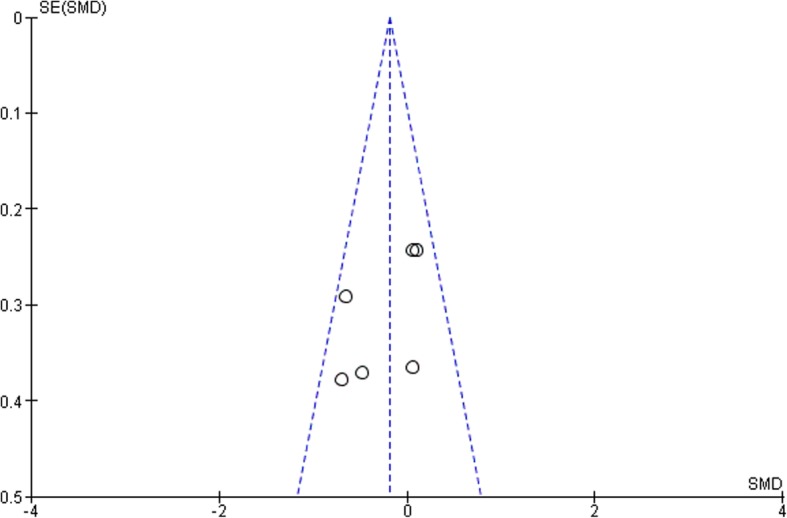
3 Month clinical attachment levels (CAL)

**Fig. 10:**
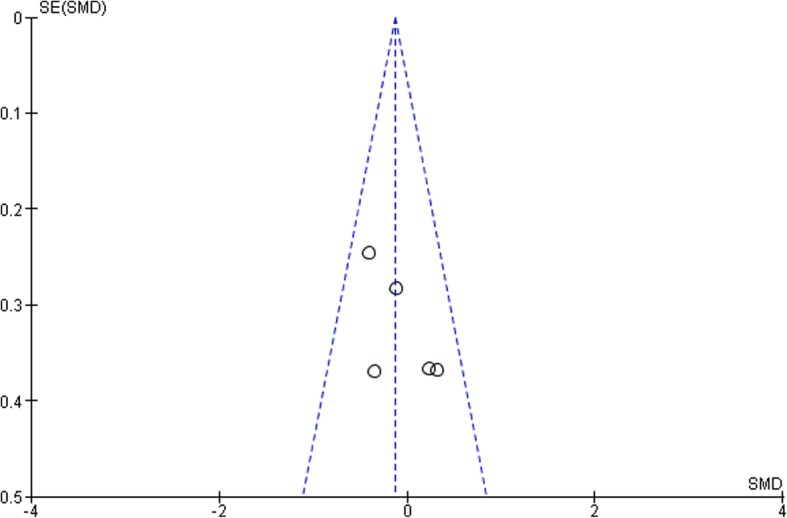
3 Month glycated hemoglobin (HbA1c)

**Fig. 11:**
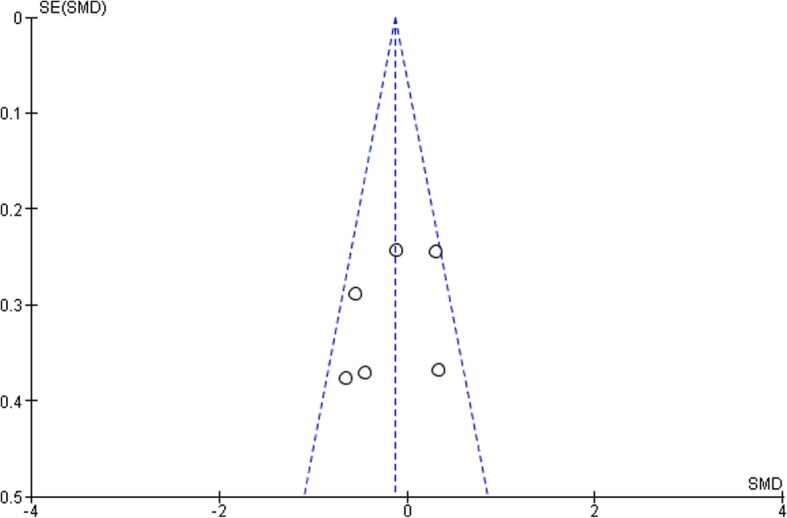
3 Month pocket depth (PD)

According to the funnel plots in Figs. 8, 9, 10 and 11, the studies involved are evenly distributed among the four funnel plots, suggesting that there was no publication bias. However, according to British Medical Journal (BMJ), tests for uneven distribution of studies in funnel plots are not used when there are 9 or less studies involved in any meta-analysis due to the low test power to differentiate substantial asymmetry from pure coincidence [26].

The main reason a meta-analysis is done is due to the fact that it has the superiority of having a better statistical power and its value as an evidence-based resource able to extrapolate confirmatory data analysis across the affected population. Nonetheless, the inadequate number of evidence in the present meta-analysis may not add to the current clinical guidelines with confidence. The limitations of this systematic review includes a lack of timeframe which only includes a 3-month follow-up after systemic doxycycline is used [7, 10, 11, 16, 27, 28].

Besides that, another issue is the allocation concealment in which the risk of bias is fully unclear. No information was given on the allocation concealment, which affects the selection bias of all the six studies. Another concern is that the definition used in the present study lacked radiographic evidence of bone loss and were single-point in time measurements, although it would be a difficult task in epidemiological studies. Also, randomization of participants were not stated for three of the studies, namely Gaikwad et al., O’Connell et al. and Al-Nowaiser et al. [10, 16, 27], which might lead to selection bias. Strength of the reviews include prior protocol registration, subgroup and sensitivity done, grade shows the quality of evidence.

## Conclusion

To sum up, systemic doxycycline as an adjunct to scaling and root planing does not significantly improve clinical attachment levels for periodontal status as well as reduction of HbA1c levels in treatment of diabetic patients with periodontitis. More randomized controlled trials with a larger population and longer follow-up periods are needed to come up with guidelines to treat diabetic patients with periodontitis in a more effective way than just scaling and root planing. Besides that, more work and planning needs to be done along with information given to reduce the risk of bias and prevent these methodologic shortcomings thus improving the quality of the trials done.

## Data Availability

The datasets used and/or analysed during the current study available from the corresponding author on reasonable request.

## Abbreviations

BOP: Bleeding on probing
CAL: Clinical attachment levels
HbA1c: Glycated hemoglobin
PD: Pocket depth
